# Consumption of energy drinks among youth in Spain: trends and characteristics

**DOI:** 10.1007/s00431-025-06177-7

**Published:** 2025-05-27

**Authors:** Ana Teijeiro, Mónica Pérez-Ríos, Guadalupe García, Lucia Martin-Gisbert, Cristina Candal-Pedreira, Julia Rey-Brandariz, Carla Guerra-Tort, Leonor Varela-Lema, Nerea Mourino

**Affiliations:** 1 Department of Preventive Medicine and Public Health, University of Santiago de Compostela, C/San Francisco s/n, 15782 Santiago de Compostela, Galicia Spain; 2 Health Research Institute of Santiago de Compostela (Instituto de Investigación Sanitaria de Santiago de Compostela-IDIS), Santiago de Compostela, Galicia Spain; 3 Consortium for Biomedical Research in Epidemiology and Public Health (CIBER de Epidemiología y Salud Pública-CIBERESP), Madrid, Spain; 4 Escola Universitaria de Enfermaría, Universidade da Coruña, 15071 A Coruña, Galicia Spain

**Keywords:** Energy drinks, Prevalence, Consumption, Spain, Adolescents, Behavior

## Abstract

The aims of this study were to describe the trend across 2014–2023 in the prevalence of the consumption of energy drinks among students aged 14 to 18 years in Spain and the shifting characteristics of consumers. Data source was the microdata of the Survey of Drug Use in Secondary Education in Spain (2014–2023). Prevalence of energy drink consumption was calculated overall, by sex and age, and in the 18 Autonomous Regions. To identify the factors linked to consumption, multivariate models were fitted. Prevalence and adjusted odds ratios are shown with their 95% confidence intervals; the geographic distribution of energy drink consumption was plotted on maps. The prevalence of consumption of energy drinks in the last 30 days ranged from 40.4% (2014) to 47.7% (2023). For any given year, prevalence was higher in boys and varied among regions. Being male, non-Spanish, a repeat student of 1 or more academic years, having no parent with higher education, or consuming tobacco, cannabis, or alcohol increased the likelihood of energy drink consumption.

*Conclusions*: The prevalence of energy drink consumption among Spanish students aged 14–18 years has shown an upward trend among both sexes, especially among girls. The characteristics of consumers have been stable since 2014. Understanding these trends, factors associated with consumption, and the differences among regions is critical to directing efforts to educate young people about the risks involved and to prevent the initiation of energy drink consumption.

**Table:** 

**What is Known:** • *The energy drink market has grown since 2000.* • *Energy drink consumption is higher in adolescents.* • *Some European countries have taken regulatory measures, such as banning energy drink sales to minors.*	
**What is New:** • *The prevalence of energy drink consumption among Spanish adolescents has shown an upward trend in 2014–2023.* • *Prevalence was higher in boys and varied among regions.* •* The characteristics of consumers were stable during this period.*

**Supplementary Information:**

The online version contains supplementary material available at 10.1007/s00431-025-06177-7.

## Introduction

The European Commission Scientific Committee on Food defines energy drinks as a customary commercial name for beverages that contain high levels of caffeine plus specialty ingredients not commonly found in sodas and juices [1]. Most energy drinks contain about 70–80 mg of caffeine [2]. Their composition shows high quantities of sugar or artificial sweeteners, in combination with minerals, amino acids, and other additives and stimulants such as guarana, taurine, and l-carnitine [1, 3, 4]. These stimulants boost the release of reward and pleasure neurotransmitters, such as dopamine or norepinephrine, which may cause a feeling of euphoria and wellbeing, and in turn, can lead to abuse of these beverages and, ultimately, addiction [3]. Although the short- and long-term effects of energy drinks remain uncertain, their consumption has been associated with health problems due to caffeine overdoses and other caffeine boosters like taurine, glucuronolactone, and guarana. Some of these health problems include cardiovascular complications, gastrointestinal upsets, psychomotor agitation, anxiety, restlessness, and insomnia [5]. Furthermore, the high sugar content in most of these beverages raises concerns about potential long-term consequences, including the risk of obesity and diabetes [6].

Energy drinks first appeared in the United States of America in 1949 with the marketing of “Dr. Enuf” [7]. They subsequently arrived in Europe in 1987 with the launch of Red Bull in Austria [8]. Since 2000, the energy drink market has grown exponentially [4], a phenomenon associated with aggressive marketing campaigns in which these drinks are promoted as boosters of physical and mental capacities [9]. These effects appeal to adolescents seeking to enhance their performance in physical and academic activities [3, 5]. Currently, energy drink manufacturers are diversifying their product lines through the launch of new varieties and flavors, with the aim of attracting new users and differentiating themselves from their competitors [10]. Moreover, access to these drinks is becoming increasingly easier for teenagers.

In a study carried out in 16 European countries in 2013, Zucconi et al. [11] estimated the prevalence of energy drink consumption in the last year at 68% in European teenagers. This consumption was higher than that of children and adults, the other two population groups studied. There are no other sources of energy drink consumption data at the European level to corroborate this information.

Since then, some European countries, such as Lithuania and Latvia, have taken proactive regulatory measures to address concerns about energy drinks, including banning sales to minors and implementing advertising restrictions, in 2014 and 2016, respectively [12–14]. Currently, Norway and Poland are in view of including legislation to restrict sales to individuals under the age of 16 and 18, respectively, with a strong focus on protecting children and adolescents [15, 16].

Given these initiatives, Spain must examine energy drink consumption trends and user characteristics to develop targeted information campaigns and adopt effective regulations. Prior studies have linked energy drink consumption to a younger age, male gender, psychoactive substance use, and low socioeconomic status [17–21].

This study seeks to enhance understanding of these characteristics and estimate their contribution to overall consumption among Spanish students. Specifically, it aims to describe the trend over time in the prevalence of energy drink consumption among students aged 14 to 18 years in Spain and its Autonomous Regions (ARs), and to identify how the characteristics of users have shifted from 2014 to 2023.

## Material and methods

### Data source and sample size

The data for this study were sourced from the microdata of the Survey of Drug Use in Secondary Education in Spain (*Encuesta sobre Uso de Drogas en Enseñanzas Secundarias en España/ESTUDES*) [22]. This survey is conducted by the Spanish Observatory on Drugs and Addictions of the Government Delegation for the National Plan on Drugs of the Spanish Ministry of Health. The aim of this survey is to ascertain the situation and trend in drug use and other addictions among Spanish students. Pupils aged 14 to 18 years, who are undergoing Compulsory Secondary Education, Senior High School Education, Basic Vocational Training, and Medium-Level Vocational Training, who are present at the day of the survey, are selected. Since 1994, this survey has been conducted biennially in schools nationwide selected by two-stage cluster sampling and collects classroom-reported data through the administration of a standardized and anonymous printed questionnaire. The first stage units are the educational centers, which are selected by applying stratified random sampling by autonomous community and center ownership (public or private). As second stage units, two classrooms per educational center are randomly selected. Data on energy drink consumption have been collected since 2014. Thus, in this study, we used data from the 2014, 2016, 2018, 2021, and 2023 surveys, which have response rates of 85.0%, 99.7%, 93.2%, 88.7%, and 86.7%, respectively.

Data on the consumption of energy drinks were gathered using the following question from the ESTUDES questionnaire: “Have you had any energy drinks (Red Bull, Burn, Monster, etc.) in the last 30 days?” (yes vs no). A user was defined as any student who answered yes to this question.

To characterize energy drink users, the following variables were evaluated: (a) sociodemographic variables, namely, sex, age, country of birth (Spain vs. others), parents’ or legal guardians’ work status (at least one gainfully employed parent—the “gainfully employed” category did not include fathers/mothers who were exclusively homemakers or house husbands, retirees, pensioners or unemployed), and educational level in the family circle (at least one parent with higher education); (b) academic performance (having vs. not having repeated at least 1 academic year); (c) students’ lifestyles, individually assessed, including smoking, cannabis use, and alcohol consumption in the last 30 days (yes vs. no). The specific questions used are included in the online resource.

As this is a national survey, pertinent regulations to conduct it have been fulfilled. In addition, according to Spanish law, parental consent is not required for participation in studies of this nature after the age of 14.

### Statistical analysis

The prevalence of energy drink consumption was calculated overall, by sex and age, and by AR (Andalusia, Aragon, Balearic Islands, Canary Islands, Cantabria, Castilla-La Mancha, Castilla y León, Catalonia, Ceuta and Melilla, Community of Madrid, Foral Community of Navarra, Valencian Community, Extremadura, Galicia, Basque Country, Principality of Asturias, Region of Murcia, and La Rioja).

To identify the factors associated with energy drink consumption, a bivariate analysis was conducted to determine which variables were linked to energy drink use (*p* < 0.2). Specifically, simple logistic regression models were used, with energy drink consumption in the last 30 days as the response variable, and each of the sociodemographic, academic performance, and lifestyle variables as predictors. Next, for each study year, a multivariate logistic regression model was fitted, including all variables that demonstrated a statistically significant relationship with energy drink consumption in the bivariate analysis. The significance level used for these analyses was 5%. Prevalence and adjusted odds ratio (OR) are shown with their 95% confidence intervals (95%CIs). To assess the geographic distribution of energy drink consumption by AR, range maps were plotted using prevalence from the first and last years (2014 and 2023, respectively), categorized in quartiles. To assess changes across the study period, we also plotted a range map showing the relative percentage change between 2014 and 2023 [((Prevalence 2023 − Prevalence 2014)/Prevalence 2014) × 100].

Calculations were performed excluding subjects with unknown values in any of the questions taken into account in the analyses.

All analyses were performed using the weighted sample and Stata v17 computer software program. The graphical analysis was performed using R software.

## Results

This study analyzed data provided by 175,394 school students in surveys conducted in 2014 (*n* = 37,486), 2016 (*n* = 35,369), 2018 (*n* = 38,010), 2021 (*n* = 22,321), and 2023 (*n* = 42,208). The characteristics of the study population are shown in Table 1 of the online resource, including unknown values. For all years except 2023, the majority of the population were girls (50.4%) and between 15 and 16 years of age (49.6%). In terms of nationality, in any given year, nine of every 10 students were Spanish nationals. The overall percentage of repeat students was approximately 20% and was higher among boys. With respect to family characteristics, the percentage of students who reported that one of their parents had undergone higher education increased with time, whereas the percentage of gainfully employed parents remained stable. When student behaviors in the last 30 days were assessed, the percentage of students who smoked was over 20% in any given year and was higher in girls; the percentage of cannabis use was over 10% and was higher in boys; and the percentage of alcohol consumption showed a downward trend, with consumption being higher in girls in all years.

**Table 1:** Prevalence of energy drink consumption (overall, by sex, and by age and sex group) in Spain for 2014, 2016, 2018, 2021, and 2023

	2014	2016	2018	2021	2023
Overall	40.4% (39.8–41.0%)	42.7% (42.1–43.4%)	40.2% (39.5–40.8%)	45.0% (44.2–45.7%)	47.7% (47.1–48.3%)
Sex					
Boys	49.7% (48.9–50.6%)	52.6% (51.7–53.5%)	49.7% (48.8–50.6%)	50.7% (49.7–51.8%)	54.4% (53.6–55.3%)
Girls	31.4% (30.6–32.2%)	32.6% (31.8–33.5%)	31.1% (30.3–31.9%)	39.0% (38.0–40.1%)	40.7% (39.9–41.6%)
Age					
14 years	36.9% (35.5–38.2%)	40.7% (39.5–42.0%)	36.9% (35.5–38.3%)	44.0% (42.4–45.6%)	42.7% (41.3–44.0%)
Boys	46.8% (44.9–48.8%)	50.5% (48.7–52.2%)	45.0% (43.0–47.0%)	46.1% (43.8–48.4%)	46.1% (44.2–48.0%)
Girls	27.8% (26.1–29.5%)	30.8% (29.2–32.4%)	29.3% (27.5–31.1%)	41.9% (39.6–44.2%)	39.4% (37.6–41.3%)
15 years	38.5% (37.4–39.7%)	44.9% (43.5–46.2%)	40.4% (39.1–41.6%)	46.0% (44.5–47.4%)	46.2% (45.0–47.3%)
Boys	48.8% (47.1–50.4%)	54.2% (52.3–56.1%)	49.4% (47.6–51.2%)	49.7% (47.7–51.7%)	52.4% (50.7–54.1%)
Girls	28.9% (27.4–30.4%)	35.0% (33.1–36.8%)	31.5% (29.8–33.2%)	42.3% (40.3–44.3%)	39.9% (38.1–41.6%)
16 years	41.9% (40.7–43.1%)	42.1% (40.9–43.4%)	40.7% (39.4–41.9%)	45.0% (43.5–46.4%)	50.0% (48.8–51.2%)
Boys	51.3% (49.5–53.0%)	52.5% (50.8–54.3%)	51.6% (49.7–53.4%)	52.3% (50.1–54.4%)	58.1% (56.5–59.7%)
Girls	33.0% (31.4–34.7%)	31.9% (30.3–33.6%)	30.6% (29.0–32.2%)	37.3% (35.4–39.4%)	41.4% (39.7–43.1%)
17 years	42.8% (41.5–44.0%)	42.0% (40.5–43.4%)	40.8% (39.5–42.2%)	44.5% (42.9–46.1%)	49.2% (47.9–50.4%)
Boys	51.2% (49.4–53.0%)	52.5% (50.4–54.6%)	51.0% (49.0–53.0%)	53.9% (51.6–56.1%)	57.2% (55.4–58.9%)
Girls	34.3% (32.7–36.0%)	31.7% (29.8–33.7%)	31.5% (29.7–33.3%)	34.6% (32.5–36.8%)	41.0% (39.2–42.7%)
18 years	43.8% (41.5–46.2%)	47.8% (44.6–50.9%)	44.9% (42.2–47.7%)	45.6% (42.8–48.5%)	52.4% (49.9–54.7%)
Boys	50.8% (47.6–54.1%)	56.0% (51.6–60.3%)	52.2% (48.5–55.9%)	50.3% (46.3–54.2%)	59.8% (56.4–63.0%)
Girls	35.4% (32.2–38.7%)	38.3% (34.0–42.8%)	36.7% (32.9–40.7%)	40.6% (36.7–44.6%)	44.2% (40.8–47.6%)

The prevalence of consumption of energy drinks in the last 30 days varied across the study period, ranging from 40.4% in 2014 to 47.7% in 2023, when it reached a peak in the study series. For any given year, prevalence was higher in boys, though the difference narrowed with time due to the rise in the prevalence of consumption among girls. In 2023, the percentage of girls who consumed energy drinks was more than 9 percentage points higher than in 2014 (31.4% vs. 40.7%), while consumption in boys remained stable. Hence, the male to female ratio was 1.6 until 2018 and dropped to 1.3 in 2023. In other words, for every female energy drink user, there were 1.3 male users. A breakdown by age showed that prevalence increased with age. For instance, in 2014, prevalence increased from 36.9% at age 14 to 43.8% at age 18. In 2021, prevalence did not change with age, remaining at around 45% for any given age (Table 1).

**Fig. 1:**
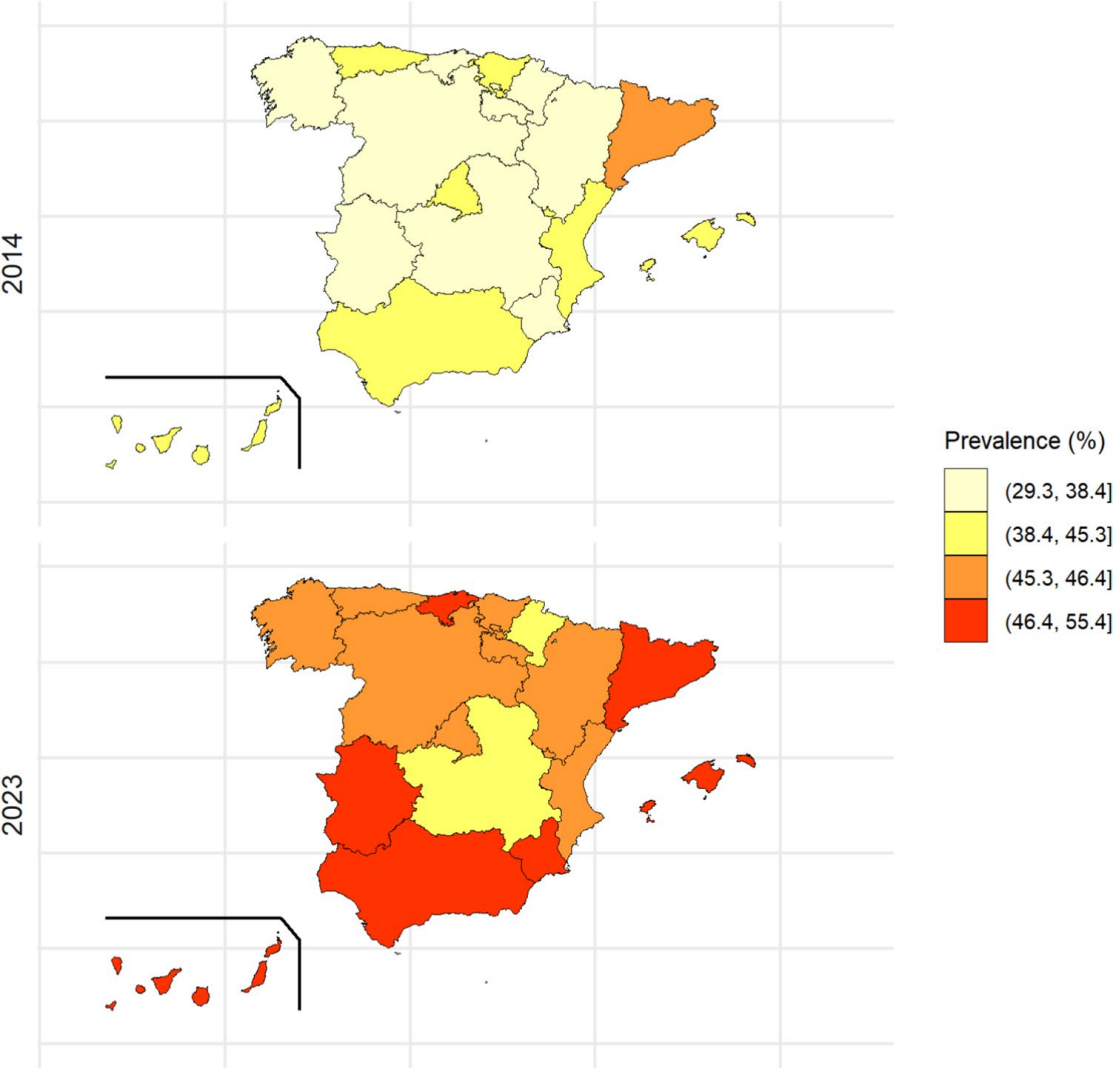
Prevalence of energy drink consumption in the last 30 days in the Autonomous Regions of Spain, categorized in quartiles, for the first and last data years (2014 and 2023, respectively)

Prevalence of energy drink consumption in the ARs ranged from 29.3 to 56.7% during the period under study. The ARs with the highest prevalences were Catalonia and the Balearic Islands in 2014. By 2023, those with the highest prevalences also included Andalusia, the Canary Islands, Extremadura, and Ceuta and Melilla (ranging from 47.5 to 55.4%) (Fig. 1).

**Fig. 2:**
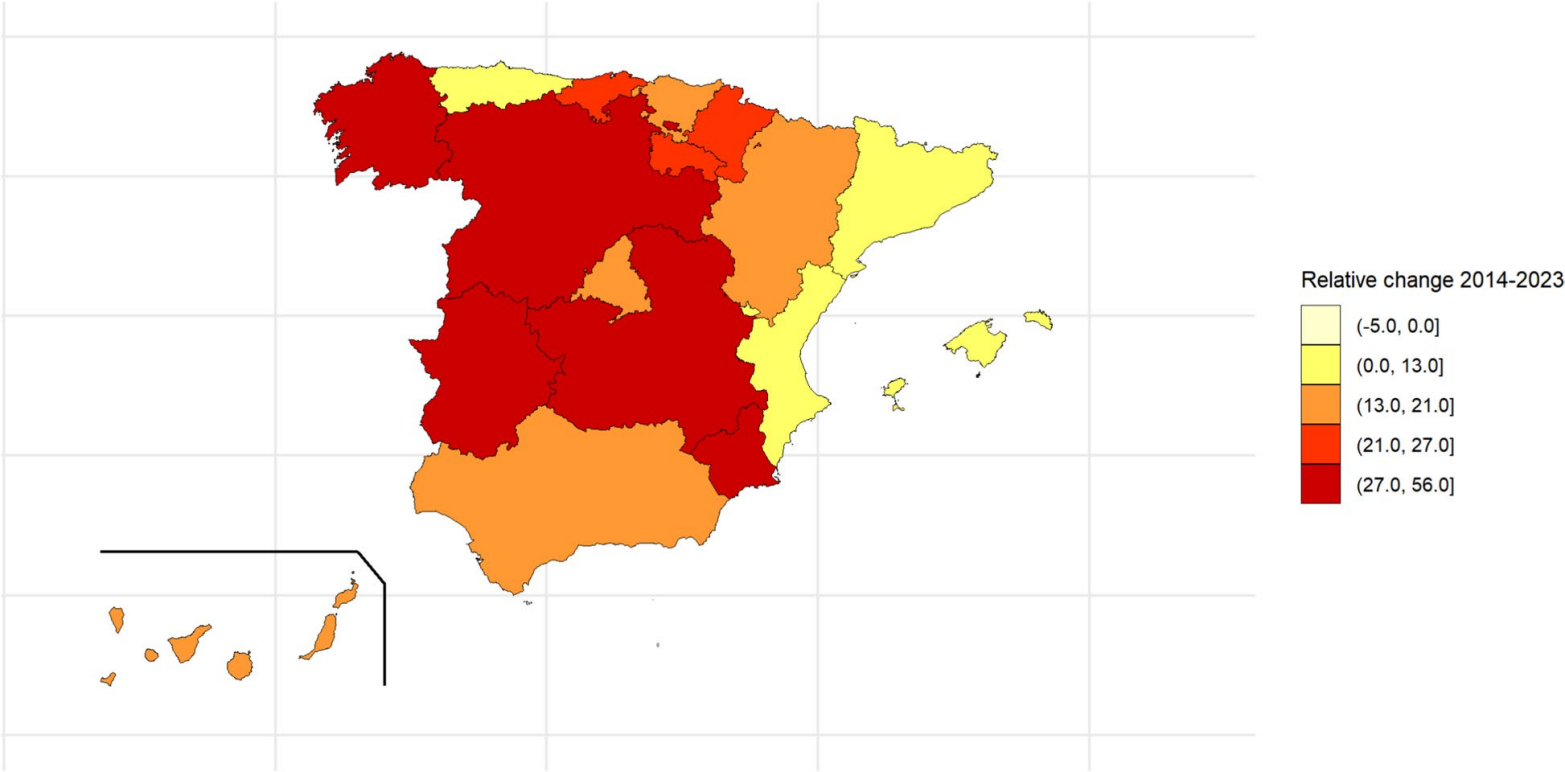
Relative change in the prevalence of energy drink consumption in the last 30 days in the Autonomous Regions of Spain for the period 2014–2023

Examination of the relative changes in the prevalence from 2014 through 2023 shows that in Ceuta and Melilla, prevalence fell across the period, and in four ARs, prevalences rose and registered relative changes from 27.0 to 56.0% (Fig. 2).

**Fig. 3:**
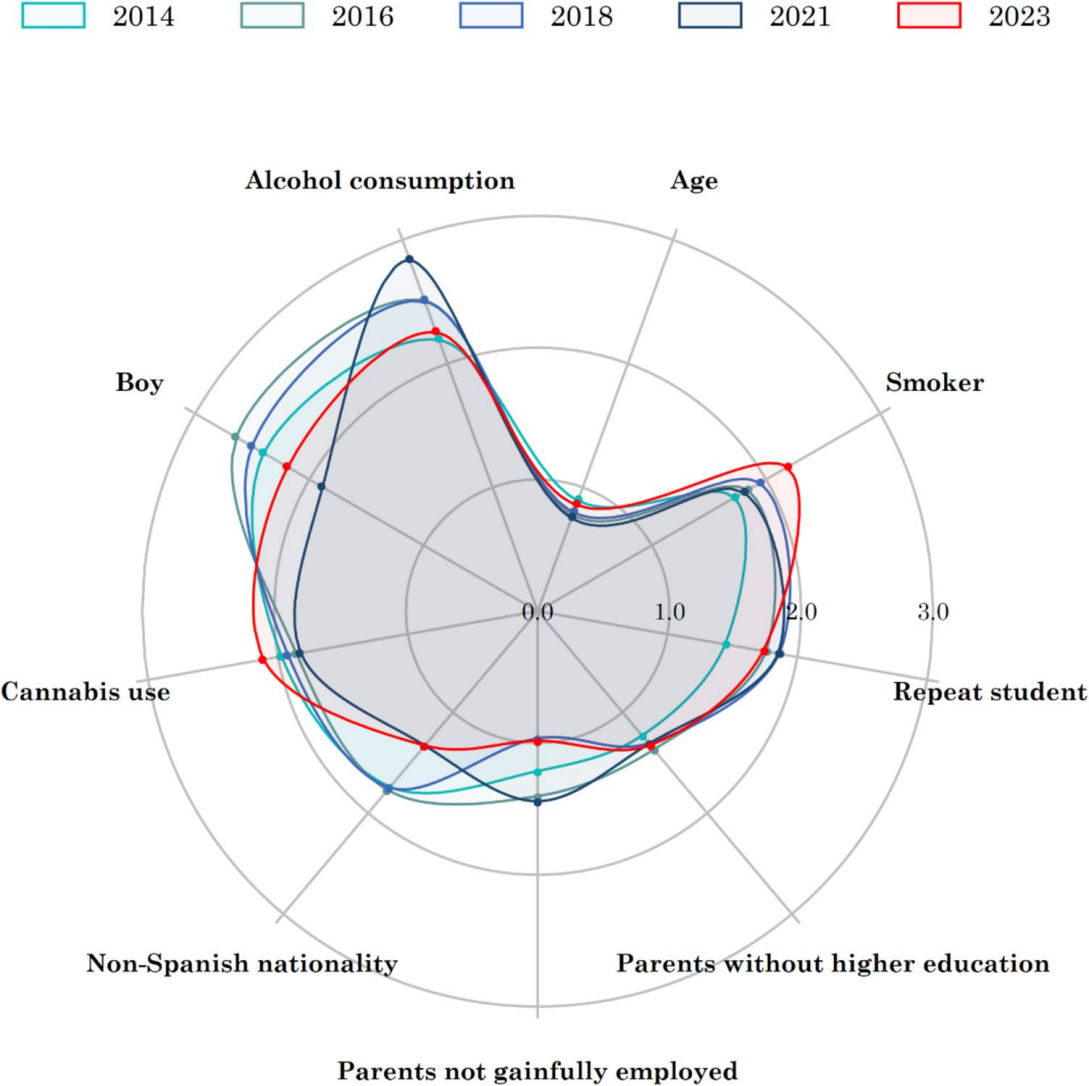
Evolution of the adjusted odds ratio of consuming energy drinks among Spanish students aged 14–18 years throughout the period 2014–2023

In any given study year, the following factors increased the likelihood of energy drink consumption: male gender, non-Spanish national status, being a repeat student of 1 or more academic years, having no parent with higher education, or use of tobacco, cannabis, or alcohol in the last 30 days (Fig. 3 and Table 2 of the online resource).

Male gender and alcohol consumption in the last 30 days were the characteristics that yielded the highest ORs. While the OR of boys consuming more energy drinks than girls decreased over time until 2021 (OR 2014, 2.4 (95% CI 2.2–2.5) vs. OR 2021, 1.7 (95% CI 1.6–1.9)), the OR of energy drink consumption increased among students who reported consuming alcohol [OR 2014, 2.2 (95% CI 2.1–2.4) vs. OR 2021, 2.8 (95% CI 2.5–3.9)] (Fig. 3 and Fig. 1 and Table 2 of the online resource).

Assessment of the changes over time in the characteristics of energy drink users showed that the OR of consumption among boys decreased with time, as did consumption among those who were non-Spanish nationals. Notably, the OR of consuming energy drinks increased among students who had smoked or used cannabis consumed tobacco or cannabis (Fig. 3 and Table 2 of the online resource).

## Discussion

The results of this study show that at least four of every ten Spanish students aged 14 to 18 years consumed energy drinks between 2014 and 2023. In general, consumption has risen over time in both sexes but is especially noteworthy in girls aged 14 and 15 years. The likelihood of consuming energy drinks is higher in students who are male, older, non-Spanish nationals, repeat students of at least 1 academic year; have fathers/mothers without higher education; and are smokers, cannabis users, or alcohol consumers.

A significant research gap exists in understanding the dynamic trends in energy drink consumption in Spain, particularly in the systematic tracking of consumption patterns and the vigilant monitoring of changes in user behavior. Our study is consistent with findings from other European countries that show an increase in energy drink consumption among adolescents. For example, a study of Finnish adolescents aged 13 and 15 found a notable increase in weekly consumption from 18.2% in 2014 to 24.4% in 2018 [23].

The case of Canada serves as a notable example that there are steps that can be taken. In 2012, regulatory changes to energy drinks required manufacturers to comply with caffeine limits, marketing restrictions, and health risk warnings [24]. This regulatory change resulted in a significant decline in energy drink consumption. In 2011, consumption estimates for the previous year were reported at 49.6% in the population aged 12–19 years and 19.1% in the previous week [25]. A subsequent study in 2017 showed a significant decline, with consumption in the previous year dropping to 32.9%, and in the previous week, to 11.8% [26]. These findings underscore the effectiveness of legislative changes as a valuable tool in promoting reductions in the consumption of such beverages among youth. In addition to these regulatory measures, role models to children and adolescents, such as athletes and influencers, given their significant public reach and potential impact on vulnerable populations, particularly children, should also bear responsibility for preventing the initiation of consumption by not participating in the promotion of energy drinks.

It is important to note that several European countries, including Lithuania, Latvia, Norway, and Poland, have also implemented similar restrictions on sales and advertising to minors. Considering the success of ongoing initiatives, which have demonstrably reduced energy drink consumption among youth, Spain should strongly consider implementing similar legislative measures to protect its own youth population.

An examination of the addiction plans of the ARs, which aim to tackle addiction at the regional level with a focus on vulnerable groups, reveals a significant gap in coverage of energy drink consumption. Specific addiction plans targeting energy drinks are currently only active in La Rioja [27] and the Region of Murcia [28], both of which were implemented after the surveys in this study. Between 2014 and 2023, both Murcia and La Rioja experienced a significant relative increase in energy drink consumption. In addition, Galicia, which has experienced the largest increase in energy drink consumption across Spanish ARs (2014, 29.3% vs. 2023, 45.6%), plans to restrict their sale to minors and regulate advertising [29].

In 2023, Ceuta and Melilla, Catalonia, Extremadura, Andalusia, the Canary Islands, and the Balearic Islands had the highest prevalence rates of energy drink consumption. Notably, these ARs have not yet included energy drink consumption in their addiction plans. It is also noteworthy that these regions face significant challenges, such as high levels of immigration, with the exception of Extremadura [30, 31]; elevated unemployment rates, with the exception of Catalonia and the Balearic Islands [32]; and lower levels of education, with the exception of Catalonia [33], compared to other areas of Spain. Recognizing these regional differences and understanding the characteristics of users is critical to grasping the complex factors influencing energy drink consumption patterns.

This study has identified a series of factors associated with an increased OR of consumption. Although some of these factors have been identified in previous studies [18, 20, 21, 23, 34], they have never been evaluated together to assess their individual contributions to the overall risk.

The results of our study reflect that boys consume more energy drinks than girls, which is consistent with the findings of other studies. Branco et al. propose a distinction in consumption motives based on gender: boys seek energy and physical performance enhancement, while girls consume out of curiosity [35]. This gender difference, coupled with male-targeted advertising influenced by gender socialization [20], likely contributes to the observed variation in energy drink consumption and may explain sex differences across countries. In particular, there has been a significant increase in energy drink consumption among girls over time. Analyzing prevalence trends from 2008 to 2019, an Italian study found an increase from 37.5% to 45.4% among boys and a more modest change from 19.9% to 20.3% among girls [36]. Although the change in consumption by girls is less pronounced than in our study, it represents a visible narrowing of the gender gap, driven mainly by a significant increase in consumption by girls. A possible explanation, according to Kaldenbach et al., who found the same trend, is a recent increase in the exposure, and perhaps susceptibility, of girls to energy drink marketing [37]. On the other hand, parallel trends among Finnish adolescents [36] suggest a plausible link to the escalating adoption of this behavior among girls, similar to patterns observed for behaviors such as smoking [20, 23].

Our study shows a significant association between higher energy drink consumption and academic underachievement, as reflected in students who repeated at least 1 academic year. This is consistent with similar associations reported in other Spanish studies [20, 21]. Parental employment status also plays a role, with children of non-working parents showing higher energy drink consumption. Socio-economic variables, especially those related to education level, emerge as critical factors influencing unhealthy lifestyles and reduced awareness of associated risks [20, 21].

Looking at nationality as a factor, not being born in Spain is associated with a higher OR of energy drink consumption. This association is consistent with a study conducted among young men in Switzerland, in which excessive energy drink consumption was associated with immigrant origin [34]. The proposed explanation suggests that the lifestyles of immigrants are shaped by their specific health context, possibly in combination with lower levels of education. These associations highlight the complex interplay between socioeconomic factors, educational background, and energy drink consumption patterns.

Furthermore, energy drink consumption was found to be correlated with tobacco use, alcohol consumption, and cannabis use in the previous 30 days. This concurrent use has been consistently reported in previous studies [18, 20]. A study conducted among students in Spain concluded that energy drink consumption was associated with increased tobacco use, alcohol consumption, and accident frequency [20]. Future studies could further explore these associations, considering the frequency of energy drink consumption and its links to other risky behaviors, as well as the potential health effects of concurrent use.

This study has some limitations. First, a limitation of the present study is the restriction of the time frame of energy drink consumption to the previous 30 days, as this is the only question regarding these beverages in the ESTUDES questionnaire. This is a limitation shared by many previous studies [38]. The paucity of uniform criteria on the methodology for the assessment of energy drink consumption is evidenced by numerous systematic reviews [38, 39]. This is an aspect that should be taken into account in future studies, as it is not possible to differentiate different consumption patterns within this time frame. Another related limitation is that we did not consider the frequency of consumption when estimating its prevalence or analyzing the factors that could influence consumption. Two recent systematic reviews emphasize that daily consumption is associated with adverse health effects, while those from occasional consumption remain less clear [40, 41]. In this regard, future research examining the health effects of energy drink consumption should account for both the frequency of consumption and the potential confounding influence of dietary patterns. The examination of variables associated with energy drink consumption is limited, precluding an assessment of the impact of factors such as personal relationships with parents or peers, as well as other lifestyle-related variables like sports performance or problematic internet use. This limitation is linked to the absence of data specifically addressing sports and internet use throughout all the periods covered by the study surveys. Additionally, the prevalence obtained at an AR level could not be analyzed by sex and age, due to the limited sample size in each AR. Finally, the data analyzed were drawn from self-reported behavior in the educational setting; this may be associated with social-desirability bias, in that students may conceal consumption, yet this aspect may well seem somewhat irrelevant in the case of energy drinks, given that they are not illegal beverages or considered harmful by adolescents [42]. The use of an anonymous, printed questionnaire might also help mitigate this potential bias.

In terms of strengths, our study utilizes a nationally representative sample that spans eight years, providing comprehensive insights into energy drink consumption trends. In particular, despite the existence of other studies with similar characteristics, this study is the first to evaluate the temporal evolution of consumption, taking into account the characterization of users. Furthermore, stratification by sex and age is emphasized, which may offer pertinent information, particularly in light of the observed differences in energy drink consumption by sex. Finally, this study provides prevalence data specific to each AR in Spain, contributing to a more nuanced understanding of regional differences in energy drink consumption patterns.

## Conclusions

The prevalence of energy drink consumption among Spanish students aged 14–18 years has shown an upward trend from 2014 to 2023 among both sexes, especially among girls. The likelihood of consuming energy drinks during this period was higher in older non-Spanish male repeating students who have parents without higher education and are smokers, cannabis users, or alcohol consumers. Importantly, these characteristics have remained stable since 2014. Recognizing and understanding these trends and factors is critical to directing efforts to educate young people about the risks involved and to prevent the initiation of energy drink consumption. This nuanced analysis, which takes into account differences between ARs, is important for informed decision-making in the formulation of policies aimed at mitigating potential health risks associated with energy drink consumption among adolescents.

## Supplementary Information

Below is the link to the electronic supplementary material.

Supplementary file1 (DOCX 219 KB)

## Data Availability

No datasets were generated or analysed during the current study.

## Abbreviations

AR: Autonomous Regions
ESTUDES: Survey of Drug Use in Secondary Education in Spain (*Encuesta sobre Uso de Drogas en Enseñanzas Secundarias en España)*
OR: Odds ratio
