# Supplementary material for: Consumption of energy drinks among youth in Spain: trends and characteristics
Source: Eur J Pediatr. 2025 May 27;184(6):365. doi: 10.1007/s00431-025-06177-7 (PMC12116719; doi:10.1007/s00431-025-06177-7)

**Online resource**

**CONSUMPTION OF ENERGY DRINKS AMONG YOUTH IN SPAIN: TRENDS AND CHARACTERISTICS THAT INFLUENCE THEIR CONSUMPTION.**

**Authors:** Ana Teijeiro,^a^ Mónica Pérez-Ríos,^a,b,c^ Guadalupe García,^a,b^ * Lucia Martin-Gisbert, ^a,b^ Cristina Candal-Pedreira,^a,b^ Julia Rey-Brandariz,^a,b,c^ Carla Guerra-Tort,^a^ Leonor Varela-Lema,^a,b,c^ Nerea Mourino, ^d^

**Author affiliations**

1. Department of Preventive Medicine and Public Health, University of Santiago de Compostela, Santiago de Compostela, Galicia, Spain
2. Health Research Institute of Santiago de Compostela (Instituto de Investigación Sanitaria de Santiago de Compostela-IDIS), Santiago de Compostela, Galicia, Spain.
3. Consortium for Biomedical Research in Epidemiology and Public Health (CIBER de Epidemiología y Salud Pública-CIBERESP), Madrid, Spain.
4. Universidade da Coruña, Escola Universitaria de Enfermaría, Oza, A Coruña, España

**Address for correspondence*:**

Guadalupe García

Department of Preventive Medicine and Public Health

C/ San Francisco s/n, University of Santiago de Compostela

15782, Santiago de Compostela, Spain.

Tel.: +34881812278

Email: [guadalupe.garcia0@rai.usc.es](mailto:guadalupe.garcia0@rai.usc.es)

Orcid: 0000-0002-3529-7655

**Supplementary Table 1.-** Characteristics of the study population.

|  | **2014** | **2016** | **2018** | **2021** | **2023** |
| --- | --- | --- | --- | --- | --- |
|  | **N=37,486** | **N=35,369** | **N=38,010** | **N=22,321** | **N=42,208** |
| **Sex** | | | | | |
| **Boys** | 49.1% | 49.9% | 48.6% | 49.8% | 50.3% |
| **Girls** | 50.9% | 50.1% | 51.4% | 50.2% | 49.7% |
| **Age** | | | | | |
| **14 years** | 20.0% | 25.8% | 20.0% | 19.8% | 19.5% |
| **15 years** | 25.4% | 21.7% | 25.2% | 25.6% | 25.4% |
| **16 years** | 24.1% | 27.2% | 26.0% | 25.0% | 25.5% |
| **17 years** | 23.1% | 20.0% | 23.3% | 22.6% | 23.1% |
| **18 years** | 7.4% | 5.3% | 5.6% | 7.0% | 6.5% |
| **Nationality** | | | | | |
| **Spanish nationality** | 89.4% | 89.4% | 90.4% | 92.1% | 91.4% |
| **Non-Spanish nationality** | 10.5% | 10.3% | 9.3% | 7.6% | 8.2% |
| **Missing** | 0.1% | 0.3% | 0.3% | 0.3% | 0.4% |
| **Having repeated at least one academic year** | | | | | |
| **No** | 73.5% | 72.9% | 76.8% | 81.0% | 80.3% |
| **Yes** | 26.4% | 26.5% | 23.0% | 18.8% | 19.4% |
| **Missing** | 0.1% | 0.6% | 0.3% | 0.3% | 0.4% |
| **At least one parent with higher education** | | | | | |
| **No** | 43.2% | 39.9% | 39.2% | 34.4% | 36.6% |
| **Yes** | 34.9% | 35.9% | 45.3% | 47.6% | 48.0% |
| **Missing** | 22.0% | 24.2% | 15.4% | 18.0% | 15.4% |
| **At least one gainfully employed parent** | | | | | |
| **No** | 6.3% | 4.8% | 3.6% | 3.0% | 2.7% |
| **Yes** | 89.2% | 90.8% | 92.4% | 88.9% | 92.4% |
| **Missing** | 4.5% | 4.4% | 4.0% | 8.1% | 4.9% |
| **Alcohol in the last 30 days** | | | | | |
| **No** | 32.9% | 33.4% | 42.3% | 47.2% | 50.0% |
| **Yes** | 60.6% | 58.5% | 48.9% | 43.8% | 45.4% |
| **Missing** | 6.5% | 8.1% | 8.8% | 8.9% | 4.6% |
| **Tobacco in the last 30 days** | | | | | |
| **No** | 75.4% | 74.0% | 74.8% | 76.8% | 80.0% |
| **Yes** | 20.6% | 21.5% | 23.2% | 21.1% | 18.3% |
| **Missing** | 4.0% | 4.5% | 2.0% | 2.1% | 1.6% |
| **Cannabis in the last 30 days** | | | | | |
| **No** | 82.0% | 81.6% | 81.3% | 84.5% | 84.3% |
| **Yes** | 12.8% | 12.3% | 13.6% | 10.5% | 8.8% |
| **Missing** | 5.3% | 6.1% | 5.1% | 4.9% | 6.8% |

**Supplementary Table 2**.- Adjusted odds ratio of consuming energy drinks among Spanish students aged 14-18 years through the period 2014-2023.

|  | **2014** | **2016** | **2018** | **2021** | **2023** |
| --- | --- | --- | --- | --- | --- |
| **Sex** | | | | | |
| **Boys** | 2.4 (2.2-2.5) | 2.5 (2.4-2.7) | 2.4 (2.3-2.3) | 1.7 (1.6-1.9) | 2.2 (2.0-2.3) |
| **Girls** | 1 | 1 | 1 | 1 | 1 |
| **Age (continuous)** | 0.9 (0.9-0.9) | 0.8 (0.8-0.8) | 0.8 (0.8-0.8) | 0.8 (0.8-0.8) | 0.9 (0.8-0.9) |
| **Nationality** | | | | | |
| **Spanish nationality** | 1 | 1 | 1 | 1 | 1 |
| **Non-Spanish nationality** | 1.7 (1.6-1.9) | 1.6 (1.5-1.8) | 1.6 (1.4-1.8) | 1.3 (1.1-1.4) | 1.3 (1.2-1.5) |
| **Having repeated at least one academic year** | | | | | |
| **No** | 1 | 1 | 1 | 1 | 1 |
| **Yes** | 1.5 (1.4-1.6) | 1.8 (1.6-1.9) | 1.9 (1.8-2.1) | 1.8 (1.6-2.0) | 1.7 (1.6-1.9) |
| **At least one parent with higher education** | | | | | |
| **No** | 1.2 (1.1-1.3) | 1.3 (1.2-1.4) | 1.3 (1.2-1.4) | 1.3 (1.2-1.4) | 1.3 (1.2-1.4) |
| **Yes** | 1 | 1 | 1 | 1 | 1 |
| **At least one gainfully employed parent** | | | | | |
| **No** | 1.1 (1.0-1.3) | 1.2 (1.0-1.3) | 1.1 (0.9-1.2) | 1.2 (1.1-1.3) | 1.0 (0.8-1.2) |
| **Yes** | 1 | 1 | 1 | 1 | 1 |
| **Alcohol in the last 30 days** | | | | | |
| **No** | 1 | 1 | 1 | 1 | 1 |
| **Yes** | 2.2 (2.1-2.4) | 2.5 (2.3-2.7) | 2.5 (2.4-2.7) | 2.8 (2.5-3.0) | 2.2 (2.1-2.4) |
| **Tobacco in the last 30 days** | | | | | |
| **No** | 1 | 1 | 1 | 1 | 1 |
| **Yes** | 1.8 (1.6-1.9) | 1.9 (1.7-2.1) | 1.9 (1.8-2.1) | 1.8 (1.6-2.0) | 2.2 (2.0-2.4) |
| **Cannabis in the last 30 days** | | | | | |
| **No** | 1 | 1 | 1 | 1 | 1 |
| **Yes** | 1.9 (1.7-2.1) | 1.8 (1.6-2.1) | 1.9 (1.7-2.1) | 1.8 (1.6-2.0) | 2.1 (1.8-2.4) |

**Supplementary Table 3. ESTUDES questionnaire**

ESTUDES QUESTIONNAIRE

**Q1. PLEASE INDICATE IF YOU ARE MALE OR FEMALE**

- Male................................. 1
- Female ............................. 2

**Q2. WHAT MONTH AND YEAR WERE YOU BORN?** Write the month in number (if you were born in January write 01; if in February 02,...).

MONTH _ _ YEAR _ _ _ _

**Q3. IN WHICH COUNTRY WHERE YOU BORN?**

- Spain……………………………. 1
- Other country…………………2

**Q8. WHAT IS YOUR MOTHER'S AND FATHER'S EMPLOYMENT SITUATION?** Put an "X" in each column; in total you must put two "X", because there are two columns of squares.

|  | Mother | Father |
| --- | --- | --- |
| Works only on household chores |  |  |
| Works (not including housework) |  |  |
| Is unemployed |  |  |
| Pensioner or retired |  |  |
| Has already passed away |  |  |
| You don't know |  |  |

**Q9. WHAT WAS THE HIGHEST LEVEL OF EDUCATION YOUR MOTHER AND FATHER COMPLETED?** Put an "X" in each column; in total you must put two "X", because there are two columns of squares.

|  | Mother | Father |
| --- | --- | --- |
| No education or unfinished primary education (less than 6 years of completed courses) |  |  |
| Primary school completed, 6th grade EGB completed, school certificate, 6 years of courses completed. |  |  |
| 1st or 2nd year of ESO, 8th year of EGB, School Graduate, First Grade Vocational Training, 4 years or courses of secondary school. |  |  |
| 3rd or 4th year of ESO, High School, BUP, COU, Second Grade Vocational Training, 5 or more years or high school courses. |  |  |
| University studies (technical engineering, teaching, diploma, bachelor's degree, doctorate, doctorate, degree, master's degree) |  |  |
| You don't know |  |  |

**Q13. HAVE YOU EVER REPEATED A COURSE?**

- Yes, a course...........................1
- Yes, 2 or more courses ………….2
- No………………………………………3

**Q24. IN THE LAST 30 DAYS, HOW OFTEN HAVE YOU SMOKED CIGARETTES?**

- Less than one day per week ………………………….1
- Some days a week, but not daily …………………….2
- Daily ………………………………………………………….3
- Never………………………………………………………….0

**Q34. INDICATES WHETHER YOU HAVE CONSUMED ALCOHOLIC BEVERAGES**. Answer "No" if all you did was take a sip or taste someone else's drink. If you have consumed alcohol, write in the corresponding box the age when you first consumed it. Have you consumed any alcoholic beverage in the LAST 30 DAYS?

- Yes……………………….1
- No………………………..2

**Q45. HAVE YOU DRINKED ENERGY DRINKS (RED BULL, BURN, MONSTER...) IN THE LAST 30 DAYS?** Do not include sports or isotonic drinks such as aquarius, powerade, gatorade... Answer “No” if all you did was take a sip or try someone else's drink.

- Yes………………………1
- No……………………….2

**Q55. HOW MANY DAYS HAVE YOU USED HACHIS OR MARIHUANA (cannabis, weed, chocolate, joint, cost, hashish oil)?** Put an “X” in each column; in total you must put three “X”, because there are three columns of squares.

- 1 day………………………1
- 2 days…………………….2
- 3 days…………………….3
- 4-5 days………………….4
- 6-9 days………………….5
- 10-19 days……………….6
- 20-39 days……………….7
- No day…………………….0

**Supplementary Figure 1.**- Odds ratio (OR) of energy drink consumption in the last 30 days and 95% (95%CI) confidence intervals resulting from fitting logistic regression models for the years 2014, 2016, 2018, 2021 and 2023.


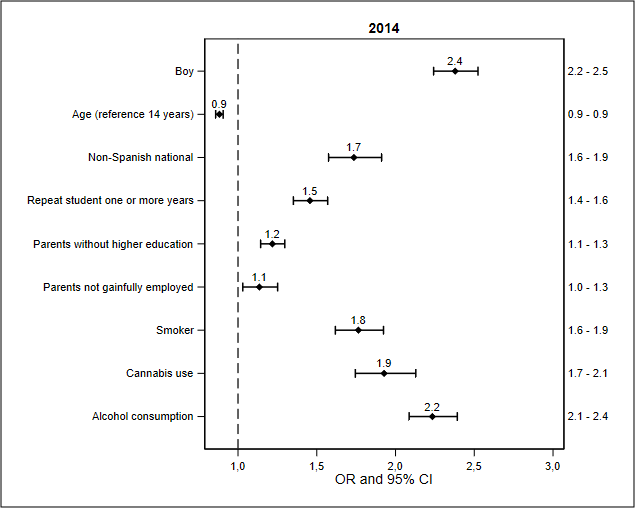


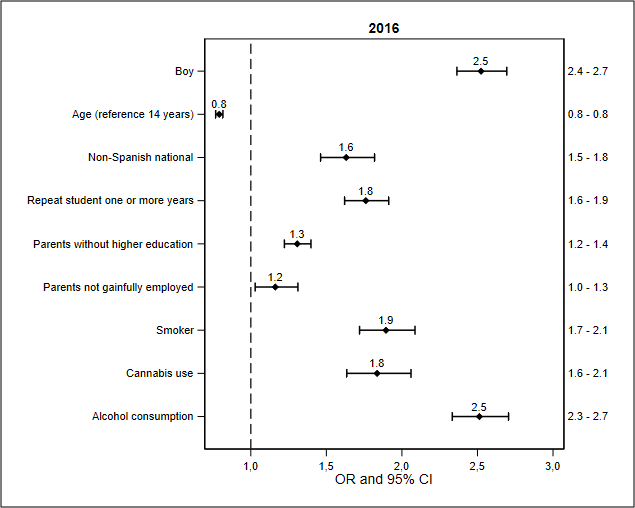


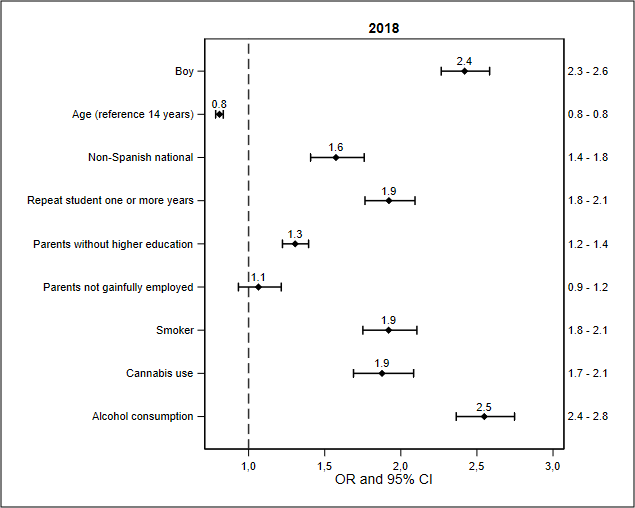


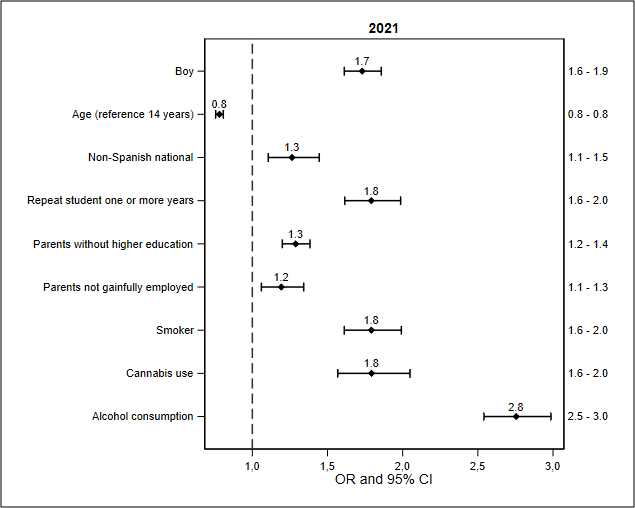


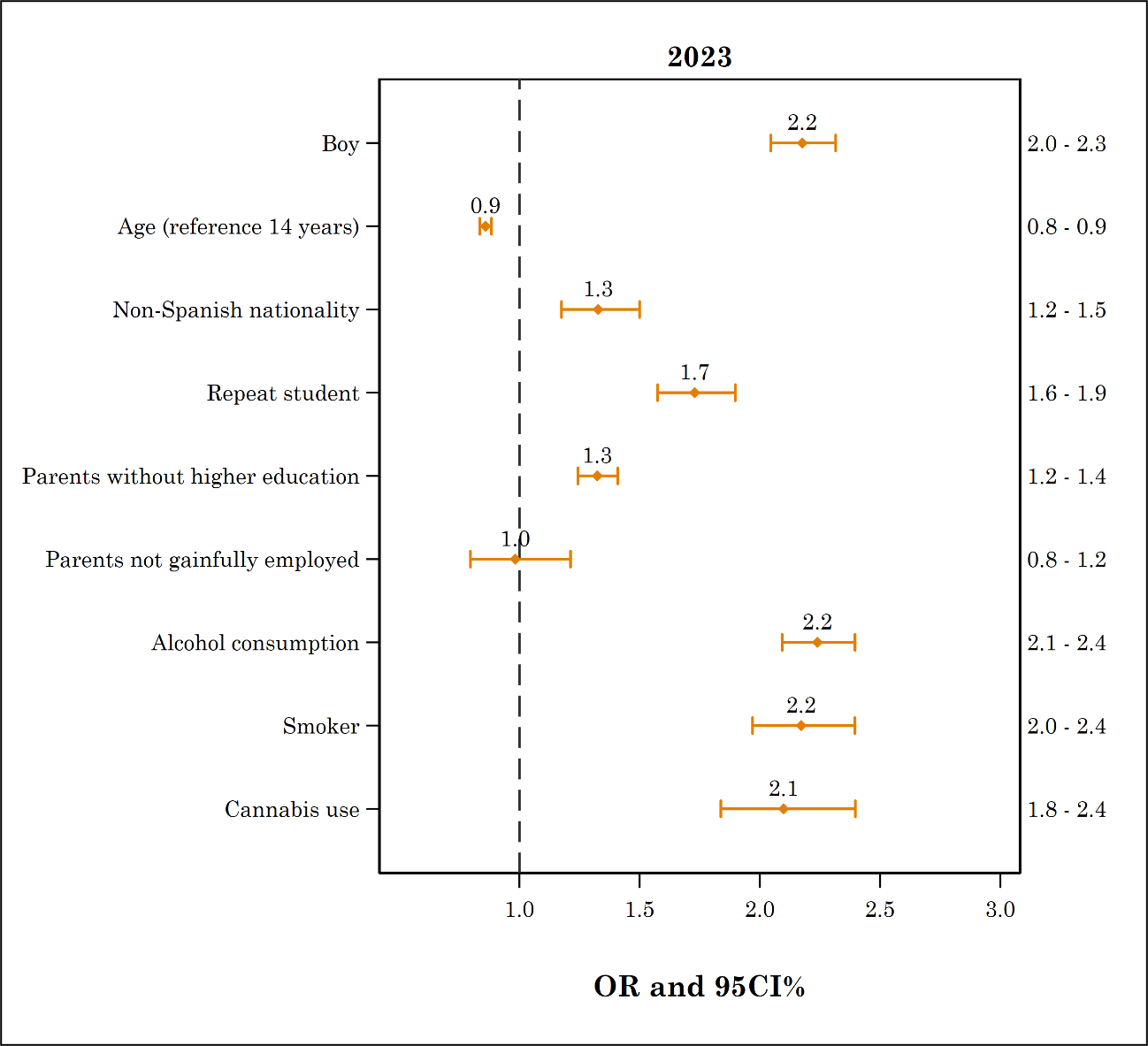

Supplement: Supplementary file 1 — Supplementary file1 (DOCX 219 KB) [file 431_2025_6177_MOESM1_ESM.docx]
